# Early detection of lung cancer

**DOI:** 10.12688/f1000research.7313.1

**Published:** 2016-04-25

**Authors:** David E. Midthun

**Affiliations:** 1 1Division of Pulmonary and Critical Care Medicine, Mayo Clinic, Rochester, MN, USA

**Keywords:** lung cancer, lung cancer screening, lung Screening

## Abstract

Most patients with lung cancer are diagnosed when they present with symptoms, they have advanced stage disease, and curative treatment is no longer an option. An effective screening test has long been desired for early detection with the goal of reducing mortality from lung cancer. Sputum cytology, chest radiography, and computed tomography (CT) scan have been studied as potential screening tests. The National Lung Screening Trial (NLST) demonstrated a 20% reduction in mortality with low-dose CT (LDCT) screening, and guidelines now endorse annual LDCT for those at high risk. Implementation of screening is underway with the desire that the benefits be seen in clinical practice outside of a research study format. Concerns include management of false positives, cost, incidental findings, radiation exposure, and overdiagnosis. Studies continue to evaluate LDCT screening and use of biomarkers in risk assessment and diagnosis in attempt to further improve outcomes for patients with lung cancer.

## Introduction

The American Cancer Society estimates that there will be approximately 224,000 new cases and 158,000 deaths from lung cancer in 2016; the current 5-year survival is about 18% ^1^. Those are sobering statistics, yet in cancers where widespread screening is employed (breast, colon, and prostate), the 5-year survival rates are significantly better: 91%, 66%, and 99%, respectively ^1^. We now know that screening for lung cancer saves lives. There is considerable relief in that statement given it has been over 30 years since the results of the Mayo Lung Project along with studies from Johns Hopkins University and Memorial Sloan Kettering Cancer Center showed lack of mortality reduction from screening with chest X-ray and sputum cytology ^2^. Advances in computed tomography (CT) technology with spiral low-dose CTs (LDCTs) allow for scanning of the entire chest in less than 15 seconds and in a single breath-hold, which is convenient and eliminates respiratory motion artefact ^3^. Early studies of screening for lung cancer with CT showed promise in detecting more cancers and more early stage cancers, and with improved survival, yet benefit in mortality reduction needed to be shown ^4– 6^. The National Lung Screening Trial (NLST) was a trial of over 53,000 high-risk individuals (defined as current smokers aged 55–74 years with 30 pack-years or if quit had done so within 15 years) randomized between screening with LDCT versus chest X-ray ^7^. The three scans (baseline and annually for 2 years) in the LDCT arm resulted in a 20% lower mortality from lung cancer ^8^. Screening may result in detection at a time when treatment is more effective and so improves outcomes and functional abilities and enhances quality of life. Implementation of LDCT screening into daily practice needs to be done with care to maximize the benefit and minimize the harms. Further studies will help better determine who to screen, how often, and how best to handle results. The potential role for biomarkers to assist or substantially redirect the lung cancer screening process is being explored.

## Screening for lung cancer: the story so far

Smoking avoidance or cessation is the primary means to prevent lung cancer ^9^. The goal for those who remain at high risk would be to detect lung cancer at an early stage, when treatment is more likely curative. In the NLST, 649 cancers were detected by CT and 367 diagnosed during follow-up post screening in the CT arm ^8^. In the chest X-ray arm, there were 279 cancers detected by chest X-ray and 525 diagnosed during follow-up post screening. Within the CT arm, 63% of lung cancers diagnosed from a positive screening test were stage I; only 29.8% were stage III or IV. Among cancers detected by chest X-ray, 47.6% were stage I, and 43.2% were stage III or IV. The reduction in advanced cancers detected with CT in the NLST demonstrated a shift in stage at diagnosis from advanced disease to early stage. After a median follow-up of 6.5 years, there were 356 lung cancer deaths among those in the CT arm versus 443 deaths among those in the chest X-ray arm, or a 20% reduction ^8^. In the NLST, the number of high-risk participants needed to screen with CT to save one life from lung cancer was 320 with three scans and 6 years of follow-up. This compares quite favorably to breast cancer, where the estimate is 781 women need to be screened for 8 years to save one life, and colon cancer where 1250 need to be screened over 8 years with fecal occult blood testing to save one life ^10^. These data indicate that CT screening is more efficient than other accepted forms of screening for cancer.

Since the publication of the findings from the NLST, screening with LDCT has been endorsed in guidelines and recommendations from various organizations including the American Cancer Society ^11^, American Lung Association ^12^, American Association of Thoracic Surgeons ^13^, American Society for Clinical Oncology ^14^, American College of Chest Physicians ^14, 15^, American Thoracic Society ^16^, National Comprehensive Cancer Network ^17^, and the US Preventive Services Task Force (USPSTF) ^18^. In 2013, the USPSTF gave LDCT screening a B recommendation (same as mammography) for screening high-risk individuals ^18^.

To date, the NLST is the only study that has shown a reduction in death from lung cancer with LDCT ( Table 1). Several other randomized CT screening trials in Europe have not shown benefit in mortality reduction, though these trials were small, underpowered, included participants at lower risk, and may have had inadequate follow-up. The Danish Lung Cancer Screening Trial (DLCST) randomized 4104 participants to CT versus no screening with lower inclusion limits of age of 50 years and 20 pack-years of smoking ^19^. After five rounds of screening, investigators reported 100 lung cancers and 39 deaths in the CT screen group versus 53 cancers and 38 deaths in the no screening group ^19^. In other words, despite CT detecting more cancers and more early stage cancers, there were comparable numbers of advanced cancers in both groups and there was no mortality reduction with CT screening. Similarly, the MILD and DANTE studies showed no reduction in mortality with CT screening compared to no screening ^20, 21^. The ITALUNG study randomized 3206 participants to LDCT versus no screening, and the Depiscan study randomized 621 participants between CT and chest X-ray and no information has been published regarding mortality in these studies ^22, 23^. The largest remaining study is the NELSON trial with 7557 participants randomized to receive CT screening, and 71% of the cancers identified were stage I and only 5% were in stage IV; information on mortality has not been published ^24, 25^. Results from the pilot of the UK Lung Cancer Screening trial are promising, showing that 35 of 42 participants (83%) found to have lung cancer on the baseline or 12-month scan underwent surgical resection ^26^. European countries have not endorsed LDCT screening at this time ^27^.

**Table 1.** Randomized controlled computed tomography screening studies.

Study	Screen modality: # Participants	Noncalcified Nodules (Baseline)	Participants with cancers	Surgical stage I	Stage IV	Deaths from Lung Cancer	Mortality Reduction
**DANTE ^21^**	**CT:** 1264	27.5%	104 (8.2%)	45%	32%	59	None
	**No screen:** 1186	NR	72 (6.1%)	22%	46%	55	
**Depiscan ^23^**	**CT:** 336	45.2%	8 (2.4%)	38%	13%	NR	NR
	**CXR:** 285	7.4%	1 (0.4%)	100%	0%	NR	
**DLCST ^19^**	**CT:** 2052	27.3%	100 (4.9%)	50%	23%	39	None
	**No screen:** 2052	NR	53 (2.6%)	15%	32%	38	
**ITALUNG ^22^**	**CT:** 1406	30.3%*	38 (2.7%)	66%	NR	NR	NR
	**No screen:** 1593	NR	NR	NR	NR	NR	
**NELSON ^24, 25^**	**CT:** 7557	50.5%	200 (2.6%)	71%	5%	NR	NR
	**No screen:** NR	NR	NR	NR	NR	NR	
**NLST ^8^**	**CT:** 26,722	27.3**	1060	50%	22%	356	20.0%
	**CXR:** 26,732	9.2	941	31%	36%	443	

**NR: not reported** * reported as positive if a nodule ≥5 mm was detected ** reported as positive if a nodule ≥4 mm was detected

How is the medical community in the US supposed to implement screening? At this point, it may be easier to come up with questions rather than the answers. Additional appropriately powered randomized studies to guide the process appear unlikely. Programs will have similar elements yet will have features that reflect local needs and resources. Policy statements and implementation reviews are available to help identify the key components of a CT screening program ^16, 28, 29^. Much of this process is being dictated by the Center for Medicare and Medicaid Services (CMS) and the American College of Radiology (ACR), which will maintain the registry through which reimbursement by CMS has been approved ^30, 31^. CMS has activated two new G codes for use for the shared decision making visit (G0296) and for the LDCT scan (G0297) and began reimbursement in January 2016 ^32^. A multidisciplinary committee consisting of pulmonology, radiology, primary care, thoracic surgery, interventional radiology, and medical and radiation oncology is important to facilitate LDCT screening, evaluate those with significantly abnormal results, and treat those with cancer. The inclusion of each of these disciplines within the process helps to assure the patient has a complete complement of options regarding diagnosis and treatment and also should limit the implementation of screening to systems with needed expertise available. Having dedicated secretarial and administrative support is important to a program’s success.

Who should be screened? A study based on the findings of the NLST found that if the screening regimen adopted in the NLST was fully implemented among screening-eligible US populations, a total of 12,250 (95% confidence interval [CI] 10,170–15,671) lung cancer deaths (8990 deaths in men and 3260 deaths in women) would be averted each year ^33^. Unfortunately, that would be fewer than 10% of the annual deaths from lung cancer. Why so few? The NLST was designed to make a small pond with big fish in it to see if fishing for lung cancer with LDCT was effective. The simplest answer of who to screen is to simply follow the NLST criteria: age 55–74 with a 30 pack-year history of smoking and either current smokers or those who have quit within 15 years ^7^. In doing so, many individuals at equivalent or higher risk than included in the NLST would be excluded from screening. There are several guidelines published recommending screening and they differ. USPSTF recommends ages 55–80 (and is so mandated within the Affordable Care Act) ^18^; CMS is reimbursing for those aged 55–77 and so defines the coverage for those in Medicare and Medicaid ^30^. The National Comprehensive Care Network (NCCN) additionally recommends screening for those aged ≥50 with a 20 pack-year history and one additional risk factor such as chronic obstructive pulmonary disease (COPD), family history of lung cancer, occupational exposure to carcinogens, and significant radon exposure ^17^. Similarly, the American Association of Thoracic Surgery recommends screening for those aged 55–79 within the NLST smoking criteria as well as those aged ≥50 with a ≥20 pack-year history and a cumulative risk of ≥5% over 5 years ^13^. At present, the American Academy of Family Physicians recommends against LDCT screening ^34^.

Age and pack-years alone do not utilize other factors know to be indicators of increased risk such as presence of COPD and a family history of lung cancer. Our program is recommending screening based on risk rather than reimbursement and, as a consequence, in addition to those who meet USPSTF criteria, recommends screening to those who have equivalent or higher risk using the PLCO _2012_ model ^35^. The primary group that this adds comprises those who smoked 30 pack-years but have quit 15 or more years ago and remain at high risk for lung cancer. In a retrospective cohort of patients who were diagnosed with lung cancer, Yang *et al.* suggest that expanding the criteria of screening to include those who quit smoking 15–30 years ago would have the potential to include 16% more of those who got cancer with acceptable cost and minimal harm ^36^.

Benefit from screening has been demonstrated in only high-risk individuals as defined by the NLST; there are no data to support screening in individuals at lower risk. There is good evidence that likelihood of benefit drops off sharply at lower risk and, as the likelihood of benefit from screening diminishes, the probability of harm increases. Within the NLST, the 60% of participants with the highest risk accounted for 88% of the prevented lung cancer deaths, while in contrast the 20% at lowest risk were the source for only 1% of the prevented lung cancer deaths ^37^. Stated another way, among those at highest risk in the NLST, only 161 participants needed to be screened for the study period to avoid one lung cancer death, while among those at lowest risk screening more than 5000 was required to save a life ^37^. Screening in lower risk individuals is to be avoided. One of the top five recommendations identified as medically appropriate and cost saving within the Choosing Wisely campaign was to avoid screening low-risk individuals for lung cancer ^38^.

Exclusion criteria should be similar between programs and include a history of lung cancer within the past 5 years, poor lung function or other serious comorbidities that would not allow one to be a candidate for surgery if needed or would greatly limit life expectancy, need for continuous oxygen supplementation, an unexplained weight loss of more than 15 lbs in the 12 months prior, recent hemoptysis, a chest CT examination in the prior 12 months, and current symptoms of an acute or resolving respiratory tract infection ^7^.

The USPSTF recommendation includes a shared decision making process (not required for breast cancer screening) ^17^; this is mandated by CMS as an identifiable visit with specific components: eligibility, absence of signs or symptoms of lung cancer, discussion of benefits and harms of screening, follow-up diagnostic testing, overdiagnosis, false positive rate, radiation exposure, importance of adherence to annual screening, impact of comorbidities, willingness to undergo treatment, and the importance of cigarette smoking abstinence or cessation ^30^. In the NLST, there were 16 deaths within 60 days of an invasive procedure and only 10 of those had cancer; patients need to know that the process of screening can be fatal ^8^. Our program mandates tobacco cessation counseling for current smokers prior to screening in an attempt to make clear that cessation is more lifesaving than screening.

The ACR and Society of Thoracic Radiology have identified specifications for LDCT and the registry requires that those technical parameters be met ^31^. A structured reporting system is desired; the ACR registry is the only approved registry and requires that the Lung Imaging Reporting and Data System (Lung-RADS) be used. Lung-RADS is only partially consistent with evidence-based guidelines, is ambiguous, and is not aimed at patient communication. Mandating a result be reported by one algorithm adds consistency and benefit if correct but stands to delay innovation to determine the best response to abnormal results. Endorsing screening in programs able to demonstrate a multidisciplinary approach may have been a better means to reduce the harms of screening rather than to focus on the radiology performance as CMS has chosen to do. Doing the CT scan is the easy part; what happens afterward is where there is opportunity to do this badly. Screening with LDCT has now shown the ability to reduce deaths from lung cancer. However, harmful effects of screening can include unnecessary testing from false positive results and incidental findings, radiation exposure, cost, biopsy and surgery for benign disease, and overdiagnosis. Other concerns include the potential for anxiety, distress, or impact on quality of life. Despite these concerns, reports have shown no significant short-term effects on quality of life in patients with LDCT screening ^39^.

## Nodule evaluation

An optimal nodule evaluation algorithm is yet to be determined and, since patient preference is to be weighed, no one fit will size all. Guidelines and nodule risk tools can assist in the decision making ^17, 40– 42^. Data show that patients nearly always assumed that their lung nodule was malignant ^43^. Given that as many as 50% of those having a screening CT will have one or more nodules detected, educating the patient about nodules and the low likelihood of malignancy is important ^5, 24^. In a survey of participants in the NELSON trial, those with a nodule detected had a short-term increase in lung cancer-specific distress, whereas those with a negative scan experienced relief ^39^. Follow-up of the CT results is imperative – a dedicated program registry is mandatory in this regard.

**Figure 1.**
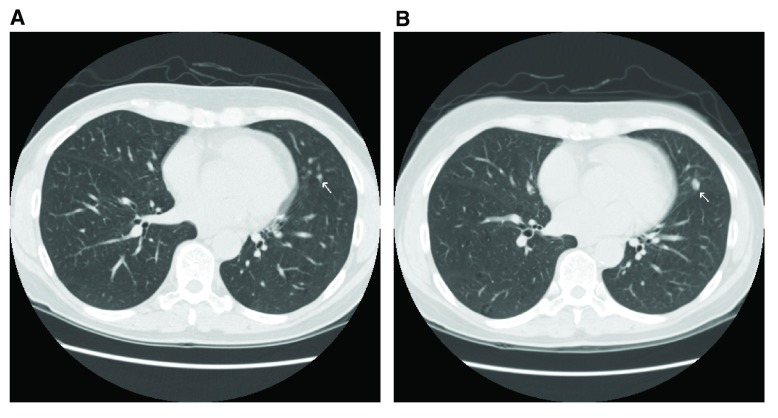
A 62-year-old woman, former smoker with a 40-pack year history, had a low-dose computed tomography (CT) screen showing a 3 mm nodule ( **A**) in the left lung (lingula). At 1-year follow-up, the nodule had grown ( **B**) and at surgical resection was a 6 mm adenocarcinoma. She remains without evidence of disease 9 years after removal.

Many raise concerns about the false positives of CT screening; within the NLST (positive defined as ≥4 mm), false positives were 96% ^8^. The 4 mm nodule has a likelihood of lung cancer of well less than 1% ^8, 42^. Should we call it a positive with that probability? The reality is that the vast majority of nodules found by CT screening need no additional evaluation other than CT follow-up – most with the next annual scan. An analysis of data from the NLST showed the percentages of lung cancer diagnoses that would have been missed or delayed and false positives that would have been avoided increased from 1.0% and 15.8% at a 5 mm threshold to 10.5% and 65.8% at an 8 mm threshold, respectively ( Figure 1) ^44^.

In a 5-year study of LDCT screening with 5203 asymptomatic high-risk individuals, primary lung cancers were detected with 77.7% being early stage and only 14.2% benign lesions diagnosed surgically ^45^. In the Mayo Clinic study, 10 (18%) of 55 participants underwent resection for benign disease ^46^. Similarly, in a German study, benign nodules represented 20% of resections ^47^. Despite narrowing what was considered a positive study, 24% and 27% of the surgical interventions in the NLST and NELSON trials, respectively, were for benign disease ^8, 24^.

Our program is recommending positron emission tomography (PET)-CT or biopsy (depending on the circumstances) only for nodules 1 cm or greater, and that eliminates immediate evaluation for over 95% of the participants. At the same time, we don’t consider a 6 mm nodule negative; it exists and needs follow-up – the key is to provide accurate information to the patient and their provider as to the likelihood of malignancy. The program is responsible for the evaluation and follow-up of findings in a desire to favorably tip the balance of benefit versus harm. People don’t die from false positives, but they can die from their evaluation. Nodule evaluation should be done by those who do it every day; this is not appropriate for the primary provider and perhaps why the American Academy of Family Practice rejects LDCT screening ^34^. Having the primary care provider evaluate the CT results would be similar to having the colonoscopist call at the time of a colonoscopy, describe the presence of an 8 mm polyp, and ask the provider, “what should I do?”

## Incidental findings

In addition to the concerns raised from abnormal opacities in the lungs, LDCT scans of the chest may find other abnormalities in the lung such as emphysema and fibrosis as well as disorders of other organs. Examples include coronary calcifications, aneurysms, nodules in the thyroid adrenals, adenopathy, and liver and kidney disease. Prevalence of incidental findings have been reported to be as high as 59–73% of those scanned ^48, 49^; clinically significant findings, defined as those requiring additional evaluation, were present on an average of 14% of those scanned ^50^. Such abnormalities may be the source of significant anxiety and uncertainty of what to do and may lead to additional testing and intervention, for which benefit has not been demonstrated.

## Radiation

CT imaging involves radiation; thus, with the chance to find lung cancer is the chance to actually induce it. Estimates of the risk of LDCT are low, even if it were performed annually over several decades. The effective dose of radiation absorption is expressed in millisieverts (mSv). The average effective dose for a standard CT of the chest is approximately 7 mSv. A low-dose scan is approximately 1.5 mSv, and this is approximately one-half of the natural ambient radiation exposure of approximately 3 mSv per year ^51, 52^. The American Association of Physicists in Medicine cites that the threshold radiation dose potentially associated with carcinogenesis is 50 mSv ^29^. The authors of the NLST estimated that the radiation risk from screening smokers aged 55 years results in one to three lung cancer deaths per 10,000 people screened and 0.3 new breast cancers per 10,000 females ^8^. This potential harm from screening highlights the importance of having proven mortality reduction through a randomized controlled trial. Whether every high-risk patient who initiates screening should continue screening annually is unclear. The NELSON trial is investigating screening at intervals of 2 and 2.5 years, and risk modeling that takes into account the findings on the baseline scan may be useful in determining for whom other than annual screening frequency is appropriate.

## Cost

With the CMS coverage determination established, it is likely that insurance companies will endorse reimbursement of annual lung cancer screenings for the appropriate populations. Cost-effectiveness analysis using data from the NLST showed that screening for lung cancer with LDCT costs $81,000 per quality-adjusted life year (QALY) gained ^53^. An actuarial modeling of LDCT using 2012 dollars estimated the cost per life year saved at $19,000 ^54^. Comparatively, annual mammography for breast cancer screening in women aged 40–80 is approximately $58,000 per QALY gained ^55^, and screening for colon cancer with colonoscopy every 10 years starting at age 50 has a cost of $56,800 per QALY gained ^56^. A projected analysis predicts that US implementation will result in 10.7 million more LDCT scans and 52,000 lung cancers detected with a total cost of $6.8 billion over a 5-year expenditure ^57^.

## Overdiagnosis

**Figure 2.**
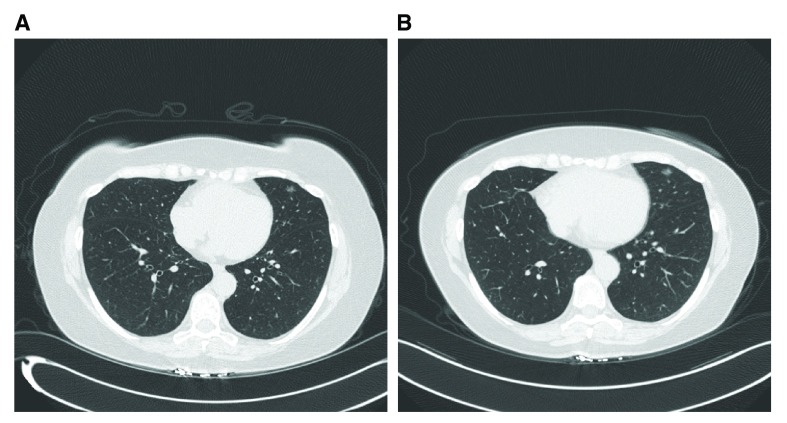
A 67-year-old woman, former smoker with a 9 mm ground glass opacity (GGO) in the lingula. The nodule has changed minimally over 3 years and is currently being followed with annual computed tomography (CT). If this is a cancer, it is likely to be an adenocarcinoma *in situ* and may represent an overdiagnosis cancer.

Overdiagnosis is recognized as a problem within lung cancer screening as it is within breast cancer and particularly prostate cancer screening. Most eventually lethal lung cancers have doubling times of 50 to 150 days, yet CT screening studies identify a subset of tumors with long tumor-doubling times of 400 days or more. These slow-growing cancers tend to appear as non-solid – either pure ground glass opacities (GGOs) or part solid nodules on CT ( Figure 2). One screening study from Japan reported tumor doubling times ranging from 662 to 1486 days with a mean of 880 days among malignancies presenting as pure GGOs ^58^. In the Mayo CT screening study, 13 of 48 (27%) screen-detected cancers exhibited doubling times that were over 400 days, suggesting these may have been overdiagnosis cancers ^59^. Knowing that mortality is reduced with screening reduces the concern for overdiagnosis, even if non-lethal cancers are detected; lives saved through screening indicate that enough fast-growing cancers were found to be of benefit. Within the CT arm of the NLST, the estimate of overdiagnosis among all cancers was 18.5% ^60^. The concept of overdiagnosis can be confusing to patients and providers, yet it is important to understand that some lung cancers can be less bio-aggressive and delay or avoidance of diagnosis and treatment may be appropriate where other clinical factors are more likely to affect life expectancy.

## Biomarkers

Given concerns over the high cost, cumulative radiation exposure, and high rate of false positives from LDCT screening, researchers are investigating the value of non-invasive biomarkers. Biomarkers may be used to evaluate risk for lung cancer, to evaluate the likelihood a nodule is a cancer, and as the primary screening test prior to the CT. Investigations have evaluated different source material such as blood, sputum, exhaled breath, and airway epithelium and with various assays such as microRNA (miRNA), methylation, antitumor antibodies, plasma proteins, airway cell, and complement fragments. Establishing utility requires validation of the biomarker in the clinical setting in which it is intended (i.e. as a predictor of the presence of malignancy in a patient with a screen-detected nodule) and should be prospectively shown to outperform other methods of evaluation.

Several trials have shown promise for using biomarkers for screening or evaluation of suspected cancer. For example, miRNAs are short single-stranded RNAs that direct the post-transcriptional repression of protein-coding genes and many are involved in oncogenesis ^61^. Investigators retrospectively evaluated plasma miRNA signatures in samples from 939 participants, including 69 patients with lung cancer and 870 disease-free individuals in the MILD screening trial ^62^. The diagnostic performance of miRNA for lung cancer detection was 87% for sensitivity and 81% for specificity; a negative predictive value (NPV) of 99% and the combination of both miRNA and LDCT resulted in a fivefold reduction of the LDCT false positive rate. Similarly, another group of investigators used a 13 miRNA signature on 1113 participants in the COSMOS lung cancer screening trial and reported sensitivity and specificity of 77.8% and 74.8%, respectively, with an NPV of 99% ^63^. A negative result was found in 810 out of the 1067 individuals without lung cancer and in 10 of the 48 individuals with lung cancer, and the authors suggest that the miRNA test could be used as a first-line screening tool.

A blood test that measures autoantibodies to lung cancer-associated antigens was tested in 1613 patients felt to be at high risk for lung cancer ^64^; 61 patients (4%) were identified as having lung cancer and 25 tested positive by autoantibodies (sensitivity = 41%). A positive autoantibody test was associated with a 5.4-fold increase in lung cancer incidence versus a negative, suggesting it may be a complementary tool to LDCT for the detection of early lung cancer. The autoantibody test is now prospectively being tested in the primary screen setting.

A validation study of a mass spectrometry, plasma-protein assay assessed the presence of malignancy in 141 CT-detected indeterminate pulmonary nodules and showed a 90% NPV and 26% positive predictive value ^65^. The results were independent of patient age, tobacco use, nodule size, and presence of COPD, suggesting proteomic classifier provides a probability estimate for the likelihood of a benign etiology in clinical assessments of pulmonary nodules.

An alternative means of predicting the likelihood that a nodule is malignant uses a gene-expression classifier measured in airway lining cells collected from the mainstem bronchus at the time of bronchoscopy. Investigators reported on 341 patients, 63 of whom had non-diagnostic bronchoscopic investigations of a peripheral nodule ^66^. The classifier had an area under the receiver-operating-characteristic curve (AUC) of 0.74, a sensitivity of 89%, and a specificity of 47%; the combination of the classifier plus bronchoscopy had a sensitivity of 98% independent of lesion size and location. The diagnostic performance of bronchoscopy was improved with the addition of the gene-expression classifier.

## Conclusion

The debate regarding the appropriate onset, interval, and benefits of screening flames on in regard to breast cancer and mammography decades after studies showed mortality reduction. Given that, we should not expect to have all the answers regarding LDCT screening at the onset of clinical implementation. LDCT screening has been shown to save lives and implementation of screening is appropriate. The goal of a CT screening program is to detect early lung cancer and facilitate curative treatment; however, primary prevention through smoking cessation or never starting is the best means to reduce the risk of dying of lung cancer. We need to get the word out to those at high risk who stand to benefit most from mortality reduction. That being said, the current USPSTF recommendations are subject to change, as many at high risk for lung cancer are excluded by the blunt assessment of age and pack-years of smoking. We want and need people to quit smoking to reduce their risk for lung cancer. The incredulity of the current recommendation is that the 55 year old, with a 30 pack-year history of smoking who does what is desired and quits smoking at onset of screening, will be told in 15 years to quit screening – when the risk of lung cancer is approximately one and a half times the risk it was when screening began ^35^. Fewer and fewer patients who actually get lung cancer are candidates for screening under the current criteria, predominantly because of the issue of stopping screening (or not starting) when beyond 15 years of having quit smoking ^36, 67^. Modifications to current screening guidelines will likely incorporate an individual’s additional factors beyond age and pack-years that increase their risk for lung cancer ^35, 68^. Risk modeling may also be used to indicate the interval for subsequent screenings based on the initial results ^69^.

Despite the focus on the radiologic performance, harm limitation centers on the appropriate evaluation of screen-detected nodules and calls for careful evaluation by pulmonologists and thoracic surgeons who do this every day and not simply passing the burden back to the primary care providers. Better methods need to be developed to separate the benign from the malignant abnormality on CT. Implementation of screening with regard to reducing harm is critical. We must implement screening in centers capable of multidisciplinary evaluation and with the expertise in dealing with lung cancer with options for video thoracic resections and reduced morbidity and options for stereotactic radiotherapy for those who may choose not to pursue surgery ^70^.

The future will likely hold use of biomarkers as an initial screening test in those at high risk and as a means of evaluating those who have had LDCT screening and have an abnormal result. No guideline presently recommends the use of biomarkers in clinical practice, but there are now biomarkers commercially available; they need to be shown to prospectively improve upon the current methods of risk assessment and diagnostic evaluation in the context of screening to be part of daily practice.
